# A strong structural correlation between short inverted repeat sequences and the polyadenylation signal in yeast and nucleosome exclusion by these inverted repeats

**DOI:** 10.1007/s00294-018-0907-8

**Published:** 2018-11-29

**Authors:** Osamu Miura, Toshihiro Ogake, Hiroki Yoneyama, Yo Kikuchi, Takashi Ohyama

**Affiliations:** 1 Department of Biology, Faculty of Education and Integrated Arts and Sciences, Waseda University, 2-2 Wakamatsu-cho, Shinjuku-ku, Tokyo, 162-8480 Japan; 2 Major in Integrative Bioscience and Biomedical Engineering, Graduate School of Science and Engineering, Waseda University, 2-2 Wakamatsu-cho, Shinjuku-ku, Tokyo, 162-8480 Japan

**Keywords:** Inverted repeat (IR), Yeast genome, IR map, Nucleosome exclusion, 3′-Untranslated region

## Abstract

DNA sequences that read the same from 5′ to 3′ in either strand are called inverted repeat sequences or simply IRs. They are found throughout a wide variety of genomes, from prokaryotes to eukaryotes. Despite extensive research, their in vivo functions, if any, remain unclear. Using *Saccharomyces cerevisiae*, we performed genome-wide analyses for the distribution, occurrence frequency, sequence characteristics and relevance to chromatin structure, for the IRs that reportedly have a cruciform-forming potential. Here, we provide the first comprehensive map of these IRs in the *S. cerevisiae* genome. The statistically significant enrichment of the IRs was found in the close vicinity of the DNA positions corresponding to polyadenylation [poly(A)] sites and ~ 30 to ~ 60 bp downstream of start codon-coding sites (referred to as ‘start codons’). In the former, ApT- or TpA-rich IRs and A-tract- or T-tract-rich IRs are enriched, while in the latter, different IRs are enriched. Furthermore, we found a strong structural correlation between the former IRs and the poly(A) signal. In the chromatin formed on the gene end regions, the majority of the IRs causes low nucleosome occupancy. The IRs in the region ~ 30 to ~ 60 bp downstream of start codons are located in the + 1 nucleosomes. In contrast, fewer IRs are present in the adjacent region downstream of start codons. The current study suggests that the IRs play similar roles in *Escherichia coli* and *S. cerevisiae* to regulate or complete transcription at the RNA level.

**Electronic supplementary material:**

The online version of this article (10.1007/s00294-018-0907-8) contains supplementary material, which is available to authorized users.

## Introduction

The multifarious structures and physical properties of DNA are thought to provide additional structural and functional dimensions to chromatin organization and gene expression (Schroth et al. 1992; Herbert et al. 1998; Liu et al. 2001; Ohyama 2001; Fukue et al. 2004, 2005; Paeschke et al. 2005; Sumida et al. 2006; Kamiya et al. 2007; Jain et al. 2008; Qin and Hurley 2008; Strawbridge et al. 2010; Du et al. 2013; Kimura et al. 2013; Nishikawa and Ohyama 2013). The occurrence of diverse DNA structures usually requires special sequence characteristics or defined symmetry elements, which are frequently found in the genomes of both prokaryotes and eukaryotes. For example, alternating purine–pyrimidine sequences, periodically occurring A-tracts, inverted repeat (IR) sequences, homopurine/homopyrimidine sequences and guanine-rich sequences lead to the formation of left-handed Z-DNAs, curved DNAs, cruciforms, triple-stranded H-DNAs (triplexes) and four-stranded G-quadruplexes, respectively (Sinden 1994). Except for curved DNAs, however, the other structures additionally require local DNA underwinding for their occurrence (Paleček 1991; Van Holde and Zlatanova 1994; Krasilnikov et al. 1999; Kouzine and Levens 2007; Sun and Hurley 2009). The dynamic processes of DNA replication and transcription generate the local DNA underwinding.

A DNA sequence that reads the same from 5′ to 3′ in each strand is known as an IR or a palindrome. IR sequences are commonly found in a wide variety of genomes, from prokaryotes to eukaryotes (Warburton et al. 2004; Wang and Leung 2009; Strawbridge et al. 2010; Cer et al. 2013; Du et al. 2013). Some of these sequences can form cruciforms with the aid of energy from negative supercoiling of DNA and in turn, cruciforms can reduce the negative superhelicity in that region (Lilley 1980; Lilley and Markham 1983; Courey and Wang 1988; Paleček 1991; Van Holde and Zlatanova 1994; Shlyakhtenko et al. 1998; Krasilnikov et al. 1999; Oussatcheva et al. 2004; Kouzine and Levens 2007). Thus, cruciforms have the potential to influence nucleosome formation and/or positioning and the local chromatin structure in eukaryotes. Numerous studies have sought to clarify the biological functions of IR sequences or cruciform structures, and suggested their participation in DNA replication (Pearson et al. 1996; Zannis-Hadjopoulos et al. 2008; Brázda et al. 2011), transcription (Dai et al. 1997; Dai and Rothman-Denes 1998; Jagelská et al. 2010; Brázda et al. 2012; Coufal et al. 2013; Miura et al. 2018), recombination (Lin et al. 1997; Shlyakhtenko et al. 2000; Lobachev et al. 2002; Wang and Leung 2006) and genome or chromosome instability (Wang and Leung 2006; Inagaki et al. 2013; Javadekar and Raghavan 2015). Furthermore, a recent study showed that short IRs with cruciform-forming potential are hotspots for genome instability in human cancer cells (Lu et al. 2015; Bacolla et al. 2016). Many reports have also suggested the presence of cruciform-binding proteins (for review, Brázda et al. 2011; Qian and Adhya 2017). However, determining the presence of cruciforms and identifying their biological role have generally been difficult, particularly in eukaryotic systems (Gentry and Hennig 2016).

With the availability of genome sequence databases, we can now easily search for IR sequences in genomic DNA. Thus, genome-wide analyses of IR sequences would provide a powerful means to assess their biological significance. Recently, genome-wide computational analyses for the distribution of IR sequences have been performed for the proteobacterium *Escherichia coli* and the budding yeast *Saccharomyces cerevisiae* (Strawbridge et al. 2010; Du et al. 2013; Miura et al. 2018). In *E. coli*, a strong enrichment of IRs with cruciform-forming potential was found in the adjacent regions downstream of the stop codon-coding sites (referred to as ‘stop codons’) and on and around the positions corresponding to mRNA ends (referred to as ‘gene ends’). Furthermore, most of the IRs with a repeat unit length of ≥ 8 bp and a spacer size of ≤ 8 bp were parts of the intrinsic terminators (Miura et al. 2018). For the *S. cerevisiae* genome, Strawbridge et al. reported that the IRs were significantly enriched and highly clustered in the intergenic regions (in this study, the genome was partitioned into coding and non-coding regions, referred to as ‘genic’ and ‘intergenic’ regions, respectively), especially in the 3′-flanking regions of the genic regions, while their occurrence in coding sequences was random (2010). These studies revealed the somewhat similar features for the occurrence of IRs or cruciform motifs between prokaryotes and eukaryotes. However, many unanswered questions still remain for the IRs in the yeast genome, including where they are located in the 3′-flanking regions of the genic regions, what primary structures they adopt, whether there is some relationship between their primary structures and positions in the genome, how these sequences influence chromatin structure in vivo, and so forth. Addressing these questions would provide clues toward clarifying the biological significance of IRs or cruciforms.

In the current study, we constructed the first *S. cerevisiae* genome-wide comprehensive map of the IRs that reportedly have a cruciform-forming potential. Furthermore, by introducing the information about the DNA positions corresponding to polyadenylation [poly(A)] sites [referred to as ‘poly(A) sites’] (i.e., gene ends), we could perform more accurate analyses than previously possible for the biological relevance of the focused IRs. We found that the IRs occur frequently in the close vicinity of poly(A) sites and ~ 30 to ~ 60 bp downstream of start codon-coding sites (referred to as ‘start codons’), and these enrichments are statistically significant. However, the effects of these IRs on the chromatin structure are different: the majority in the former regions excludes nucleosomes, while the IRs in the latter regions are incorporated into the + 1 nucleosomes. The DNA sequence analysis revealed that the enriched IRs comprise three different types: two types are in the close vicinity of poly(A) sites and another type is in the open reading frame (ORF) region. Furthermore, we found a strong structural correlation between the former two types and the poly(A) signal. Moreover, our analyses provided clues about the functions of the IRs conserved between *E. coli* and *S. cerevisiae*.

## Materials and methods

### Genome sequence and gene annotation

We obtained the full genome sequence of *S. cerevisiae* from the *Saccharomyces* Genome Database (SGD, https://www.yeastgenome.org). Gene annotations for *S. cerevisiae* were from SGD (R64) and Park et al. (2014).

### Partitioning of the genome

We defined the ‘genic’ and ‘intergenic’ regions as follows: genic: ORF, 5′- and 3′-UTRs (untranslated regions) and OUR-1, -2, and -3 (OUR: overlapping untranslated region; OUR-1, the 5′-UTR of one gene partially or completely overlaps that of another gene; OUR-2, the 3′-UTR of one gene partially or completely overlaps the 5′-UTR of another gene; OUR-3, the 3′-UTR of one gene partially or completely overlaps that of another gene); and intergenic: ‘TAN’ (the region between tandem genes), ‘DIV’ (that between divergent genes) and ‘CON’ (that between convergent genes). The information about the transcription start sites (TSSs) and the poly(A) sites for protein-coding genes was obtained from Park et al. (2014) and that about the start codons and the stop codons was obtained from the SGD. The terms ‘tandem’, ‘divergent’ and ‘convergent’ refer to the directions of transcription for the abutting genes. For intergenic regions, only those that had two clear ends, such as two TSSs, a poly(A) site and a TSS, or two poly(A) sites, were analyzed. In the cases where two protein-coding genes contain a pseudogene, tRNA gene, rRNA gene or these genes in between, the entire region between the two protein-coding genes was not subjected to further analyses.

### IR identifier

We used the computer program ‘CIRI’, which judges a given sequence as a target IR when the repeat unit length is longer than or equal to 5 bp, the spacer length is 0–8 bp and the entire IR length is longer than or equal to 13 bp (Miura et al. 2018). The CIRI program was run against the *S. cerevisiae* genome.

### Genome-wide distribution map of IR sites

The method was recently reported (Miura et al. 2018). Briefly, the location of each IR was mapped by the position of the central base pair. When an IR is located inside a larger IR, only the outer IR was used for the analyses. To construct the genome-wide distribution map of the IR sites, the Circos software (Krzywinski et al. 2009) was used. Furthermore, we developed a web-based server, ‘Cruciform-formable IRs in the *S. cerevisiae* genome (CFIRs-Sc)’ (http://www.waseda.jp/sem-ohyama/CFIRs-Sc), which is an application for browsing the map interactively.

### Regional distribution profiles of IRs

The regional distribution profiles of IRs were drawn using two homemade scripts. One sorts the IRs into the partitioned regions (ORF, 5′- and 3′-UTRs, etc.). The other measures the distance between a given IR and each end of the relevant region.

### Randomized control sequences and statistical analysis

The *S. cerevisiae* genome was partitioned into coding (ORF) and noncoding (non ORF) regions, according to its SGD annotations. The sequence randomization was performed by the method of Strawbridge et al. (2010) and Miura et al. (2018). Using 100 randomized genomes as the “control genomes”, we obtained control data. Using the test datum and the corresponding 100 control data for each bin of 10 bp, the Grubbs test was performed to examine whether the former was a significant outlier.

### Sorting of the IR sequences

Based on the AT content, the occupancy of the longest A (or T)-tract (greater than or equal to three runs of A or T) and the occupancy of the longest (ApT)_*n*_ [or (TpA)_*n*_] (*n* ≥ 1) in a repeat unit, the IR sequences were sorted into seven types (types I–VII).

### Nucleosome occupancy

The MNase-seq data were downloaded from the NCBI SRA database under the accession number SRR2045610, and processed to generate the BED files of the paired-end read data corresponding to 16 chromosomes (Ocampo et al. 2016). Using the files and the iNPS algorithm (Chen et al. 2014), the nucleosome positions in each chromosome were determined. When a given region was incorporated into a nucleosome, the nucleosome occupancy of the region was defined as 1.0 and when it was not, the value was defined as 0. The nucleosome occupancy data based on the chemical cleavage were obtained from Chereji et al. (2018).

The IRs were collected independently (IR by IR) and aligned with their center positioned at 0. Subsequently, the per-position nucleosome occupancy values were calculated and averaged from the upstream position to the downstream position. The averaged values were normalized to the average nucleosome occupancy of each chromosome that was defined as 1.0. The resulting values were abbreviated as average *nNuOcs*.

## Results

The current analyses excluded the IRs that seemed to have no potential for transition into cruciforms. To our knowledge, the shortest stem in a cruciform heretofore reported is 5 bp (Sheflin and Kowalski 1985; Iacono-Connors and Kowalski 1986; Müller and Wilson 1987; McMurray et al. 1991; Dai et al. 1997; Dai and Rothman-Denes 1998; Jagelská et al. 2010; Nuñez et al. 2015), and the typical number of nucleotides in a loop has been suggested to be 3–6 (Hilbers et al. 1985; Furlong and Lilley 1986; Gough et al. 1986; Nag and Petes 1991; Sinden 1994; Potaman and Sinden 2005). However, larger loops can also be formed in some cases, and even motifs with no spacer can form loops in the resulting cruciform (Furlong and Lilley 1986; Gough et al. 1986; Scholten and Nordheim 1986; Müller and Wilson 1987; Damas et al. 2012). Thus, we focused on the IRs with repeat unit lengths greater than or equal to 5 bp, spacer lengths between 0 and 8 bp and an entire IR length longer than or equal to 13 bp. The IRs are named and grouped in the following manner; e.g., R8S4 (the IR with repeat unit length of 8 bp and spacer length of 4 bp), for convenience. Imperfect IRs were excluded from the screening. The reasons were as follows: they occur less frequently than perfect IRs, undergo spontaneous mutations to form more perfect IRs and require higher energies for cruciform formation (Benham et al. 2002; Van Noort et al. 2003).

### Distribution of IR sequences with cruciform-forming potential

**Fig. 1:**
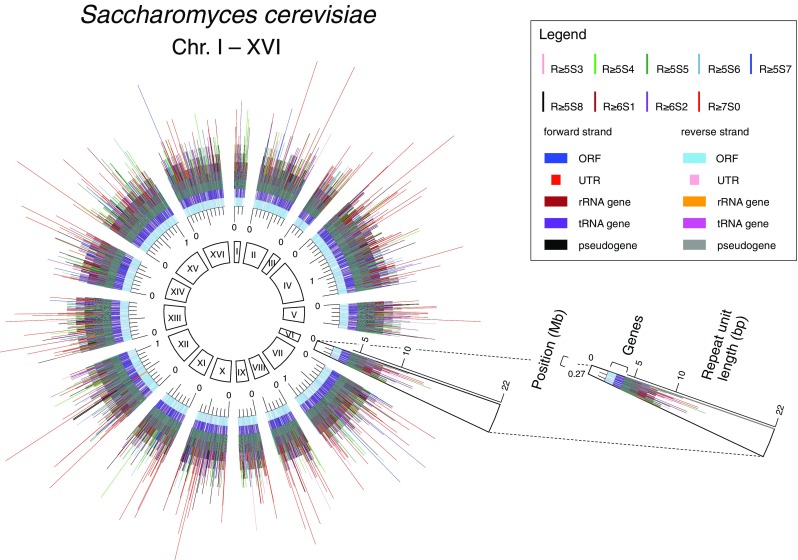
Distribution of IRs in the *S. cerevisiae* genome. The position coordinates of the R ≥ 5S ≤ 8 (2R + S ≥ 13) IRs are overlaid on the map of genes with annotations, with their repeat lengths shown as line heights. The map can also be browsed interactively in the CFIRs-Sc (http://www.waseda.jp/sem-ohyama/CFIRs-Sc)

At first, we constructed a comprehensive map for the R ≥ 5S ≤ 8 (2R + S ≥ 13) IRs with the following information: their positions and structures, genes with annotations, and positions of TSSs and poly(A) sites (Fig. 1, http://www.waseda.jp/sem-ohyama/CFIRs-Sc). Although the loci of pseudogenes and rRNA and tRNA genes are shown in the map, these were not subjected to further analyses. This is because pseudogenes generally have incomplete information for the TSS and poly(A) site, and most of the IRs detected in rRNA and tRNA gene loci are used to form the secondary structures of the corresponding RNA molecules. Thus, the analyses described below focus on protein-coding genes and their flanking regions.

**Fig. 2:**
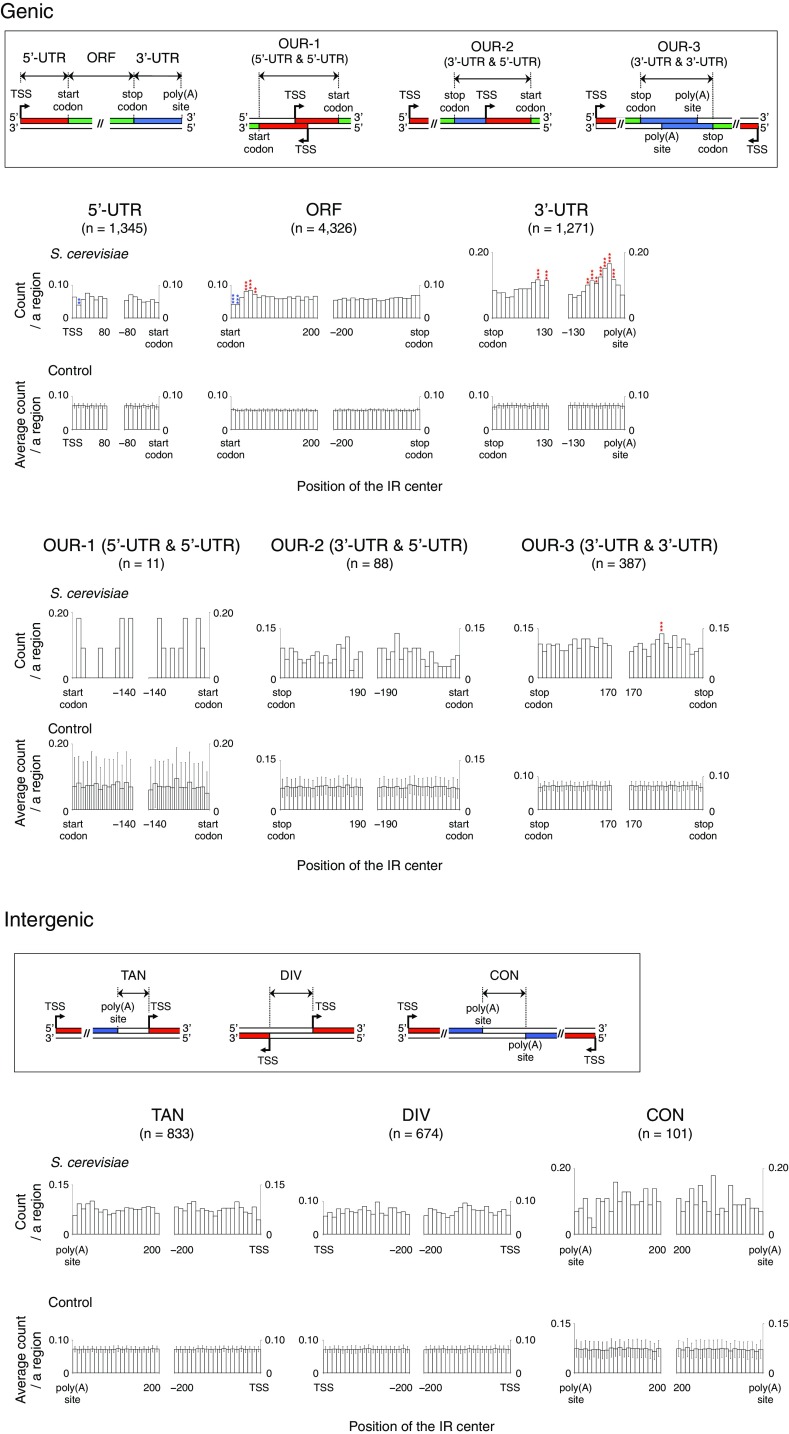
Regional occurrence frequencies of the IRs. The regional occurrence frequencies of the R ≥ 5S ≤ 8 (2R + S ≥ 13) IRs were analyzed. Genic and intergenic regions were subdivided, as schematically shown in the insets. The positions of the IRs are represented by their center positions. A TSS, the first nucleotide of a start codon, the third nucleotide of a stop codon, and a poly(A) site were each defined as position 0. In each panel, the span of the *x*-axis indicates the average length of a given region, except for the ORF, TAN, DIV and CON panels (Supplementary Table S1). The samples with lengths larger than the average length were subjected to the analysis, to obtain the information about the region that all samples have in common (‘*n*’ indicates the number of samples). The average lengths of ORFs, TANs, DIVs and CONs are 1536 bp, 305 bp, 420 bp and 209 bp, respectively, and thus only 200 bp regions from the relevant two positions were analyzed. The control data were obtained using 100 control genomes (“Materials and methods”) and the statistical significance levels were calculated based on the Grubbs test. The bin size is 10 bp. **P < 0.01, ***P < 0.001 (red, enrichment; blue, deficiency)

**Fig. 3:**
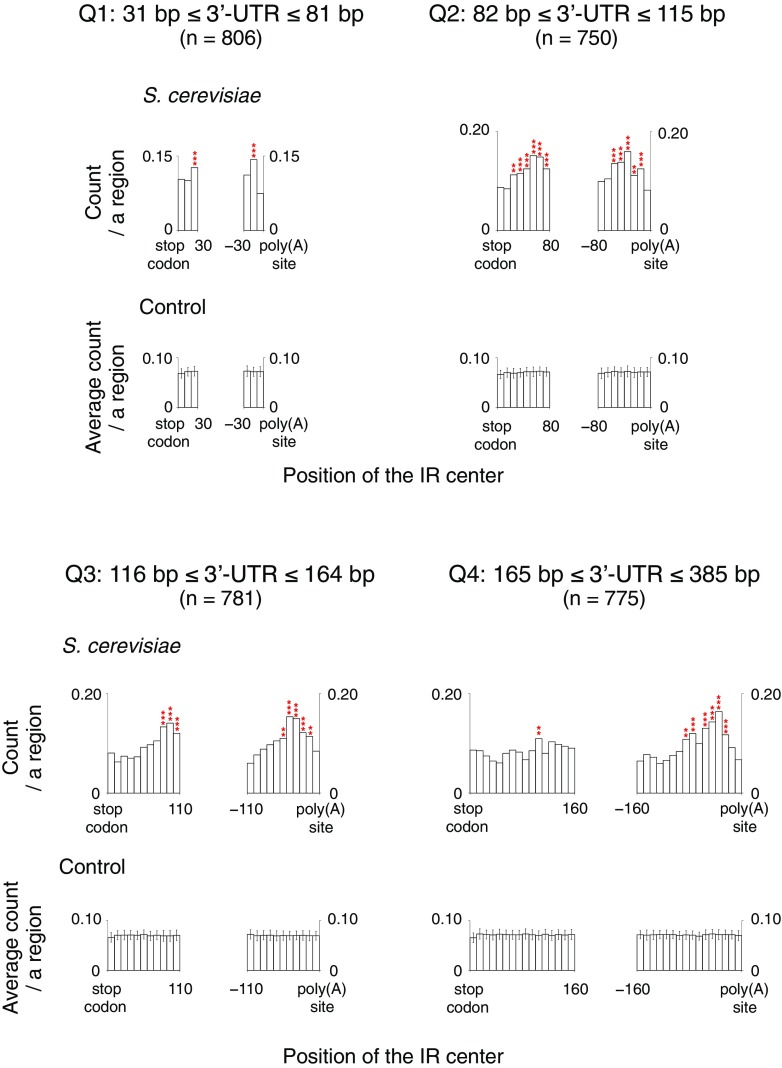
Position of the IRs in 3′-UTRs. According to the length, 3′-UTRs were sorted into five groups, and the four groups named Q1–Q4 were subjected to the analysis: Q1, 31 bp ≤ 3′-UTR ≤ 81 bp; Q2, 82 bp ≤ 3′-UTR ≤ 115 bp; Q3, 116 bp ≤ 3′-UTR ≤ 164 bp; Q4, 165 bp ≤ 3′-UTR ≤ 385 bp. In each group, the position histogram of the R ≥ 5S ≤ 8 (2R + S ≥ 13) IRs is shown. The span of the *x*-axis corresponds to the region range common among a given group. ‘*n*’ indicates the number of 3′-UTR samples. The bin size is 10 bp. **P < 0.01, ***P < 0.001 (red, enrichment)

The distribution profile of the IRs in the yeast genome shows that the IRs with a repeat unit of ≥ 10 are rare in the genome (Fig. 1, http://www.waseda.jp/sem-ohyama/CFIRs-Sc). In contrast, the IRs belonging to the R5S ≤ 8 (2R + S ≥ 13) seem to be abundant. Subsequently, we examined whether any regional characteristics are associated with the IR occurrence. For this analysis, the yeast genome was partitioned into six genic and three intergenic regions, as shown in Fig. 2. Furthermore, 100 randomized sequences were generated for each of the genic and intergenic regions (“Materials and methods”) to determine whether the apparent enrichment or deficiency of the IRs in a given region is statistically significant. The analysis showed that the IRs with cruciform-forming potential are enriched in 3′-UTRs and ~ 30 to ~ 60 bp downstream of start codons and the enrichments are statistically significant (Fig. 2). In 3′-UTRs, the regions of enrichment were ~ 20 to ~ 90 bp upstream of poly(A) sites and ~ 100 to ~ 130 bp downstream of stop codons. The data suggested that the IRs are located closer to poly(A) sites than stop codons. To confirm this, 3′-UTRs were sorted by width and the same analysis was performed, which clearly showed that the IRs are located closer to poly(A) sites (Fig. 3). Finally, we note that the distribution analysis shown in Fig. 2 also revealed that fewer IRs were present in the adjacent regions downstream of start codons and around ~ 15 bp downstream of TSSs.

### Sequence characteristics of the IRs

**Fig. 4:**
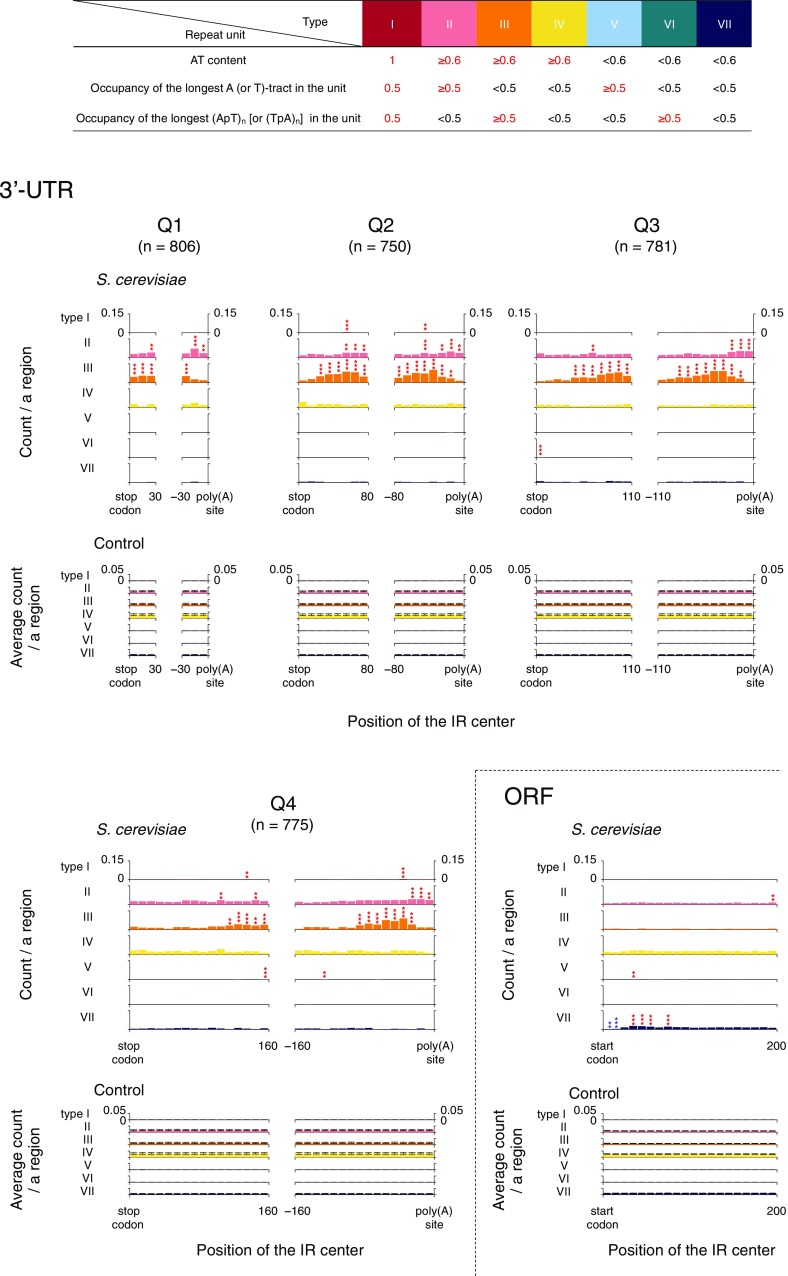
Sequence characteristics of the IRs in 3′-UTRs and ORFs. The R ≥ 5S ≤ 8 (2R + S ≥ 13) IRs were classified into seven types according to AT content, A- or T-tract occupancy and (ApT)_*n*_ or (TpA)_*n*_ occupancy in a repeat unit. The occurrence profiles of these types in 3′-UTRs and ORFs are shown. For the Q1–Q4 groups, see Fig. 3. The bin size is 10 bp. **P < 0.01, ***P < 0.001 (red, enrichment; blue, deficiency)

The sequence characteristics of the IRs located in 3′-UTRs and ~ 30 to ~ 60 bp downstream of start codons were subsequently examined. In this analysis, the sequences of the R ≥ 5S ≤ 8 (2R + S ≥ 13) IRs were sorted into seven types, according to AT content, A- or T-tract occupancy and (ApT)_*n*_ or (TpA)_*n*_ occupancy in a repeat unit (Fig. 4). Regarding the AT content, the value of 0.6 comes from that of the *S. cerevisiae* genome of 0.62. The sequence type III occurred in 3′-UTRs most frequently, and was especially eminent in the ~ − 30 to ~ − 60 region relative to poly(A) sites. The sequence type II was the second most frequent in 3′-UTRs and ~ − 10 to ~ − 20 relative to poly(A) sites were more eminent for this phenomenon. Thus, the type III IRs are generally located slightly upstream of the type II IRs. The sequence types III and II are both AT-rich (AT content ≥ 0.6), but they differ in that the former is (ApT)_*n*_ or (TpA)_*n*_-rich (≥ 0.5) in a repeat unit while the latter is A- or T-tract-rich (≥ 0.5). The sequence type I, which is somewhat similar to both types III and II, is also enriched in restricted small regions that are located within the type III and/or type II-enriched regions, although it occurs much less frequently than these types.

In the region ~ 30 to ~ 60 bp downstream of start codons, the sequence type VII, which is neither AT-rich, A- or T-tract-rich, nor (ApT)_*n*_ or (TpA)_*n*_-rich, was enriched. The sequence type V [neither AT-rich, (ApT)_*n*_-rich nor (TpA)_*n*_-rich] is also enriched in a restricted small region within the type VII-enriched regions, although its occurrence frequency is much lower than that of type VII.

### Localizations of the IRs in chromatin

**Fig. 5:**
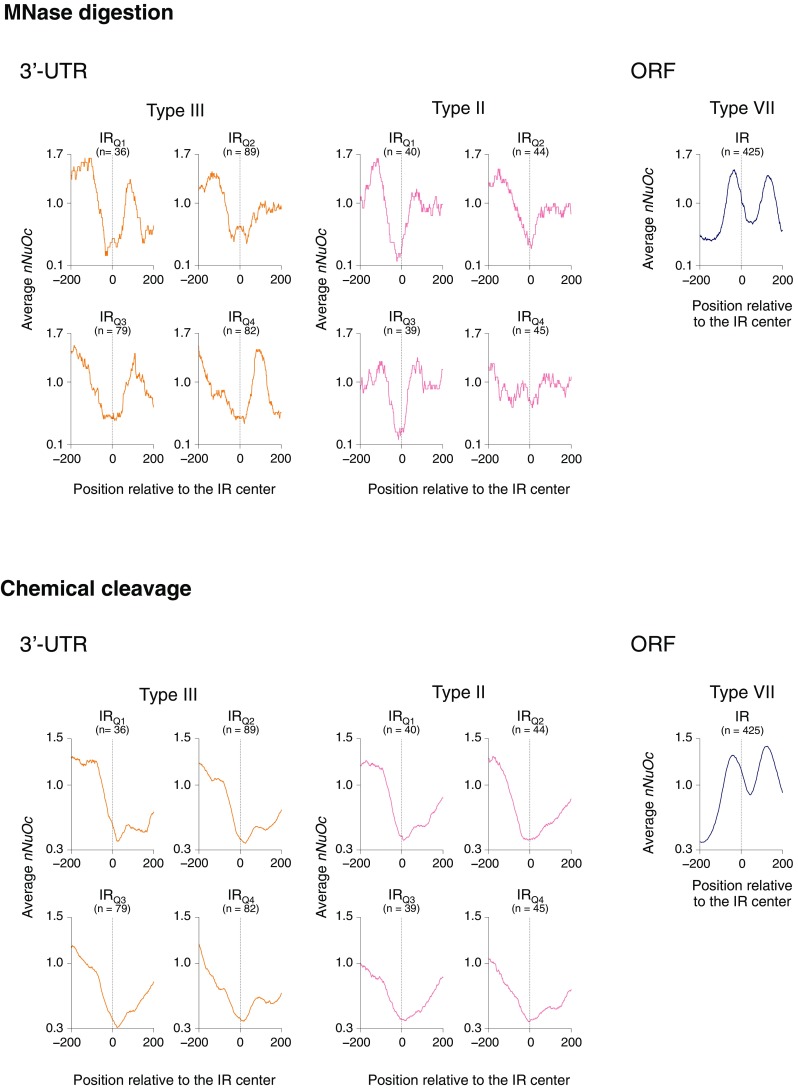
Nucleosome occupancy on and around the IRs. The IRs (types III and II for 3′-UTRs and type VII for ORFs) that showed statistically significant scores for the occurrence (Fig. 4) were subjected to the analysis. IR_Q1_–IR_Q4_ mean the IRs located in the 3′-UTR length groups Q1–Q4 (Fig. 3), respectively. The average *nNuOc* value (“Materials and methods”) for each base pair located from − 200 to + 200 relative to the IR center, indicated as 0, was calculated and plotted. In the case of tandem genes, the low nucleosome occupancy on the promoter of the downstream gene may affect the total profile. Thus, only convergent genes were used in this analysis. The data of nucleosome positions were obtained from Ocampo et al. (2016) (based on MNase digestion) and Chereji et al. (2018) (based on chemical cleavage)

Cruciform structures are incompatible with nucleosome structures (Nickol and Martin 1983; Nobile et al. 1986; Battistoni et al. 1988; Van Holde and Zlatanova 1994; Pearson et al. 1996). Accordingly, we can roughly speculate on the potential of a given IR to transition into a cruciform in vivo, by examining where it is located in the chromatin. Several groups have reported genome-wide nucleosome maps for budding yeast (Kaplan et al. 2009; Brogaard et al. 2012; Henikoff et al. 2014; Hu et al. 2014; Ramachandran et al. 2015; Ocampo et al. 2016; Chereji et al. 2018). At first, we used the MNase-seq-based map of Ocampo et al. (2016) for this purpose. This map is based on the paired-end sequencing, which provides more accurate nucleosome positions than single-read data (Cole et al. 2012; Ocampo et al. 2016). For the chromatin of 3′-UTRs, the positions of the types III and II IRs were examined and for those formed ~ 30 to ~ 60 bp downstream of start codons, the type VII IRs were examined. As shown in Fig. 5, a clear difference was found between the two results. In the chromatin of 3′-UTRs, the types III and II IRs are generally located at the bottom or very close to it in each profile, indicating that these types are more preferentially located in the linker DNA regions than the other DNA sequences in 3′-UTRs. Furthermore, the profile in each panel is asymmetric and the peak appearing on the upstream side is generally higher than that on the downstream side, indicating that the nucleosome occupancies generally differ between upstream and downstream of the IRs. In contrast, for the chromatin formed on the ~ 30 to ~ 60 bp downstream region of start codons, the majority of the type VII IRs is located within nucleosomes, which are most certainly the + 1 nucleosomes (Tirosh et al. 2010; Tsui et al. 2011).

As an alternative to drawing nucleosome maps, chemical cleavage-based methods are known, and they can reportedly avoid the cleavage bias caused by the preference of MNase for A/T-rich regions and be thought to provide more accurate data on nucleosome positions (Brogaard et al. 2012; Henikoff et al. 2014; Chereji et al. 2018). Thus, using the chemical cleavage-based nucleosome map of Chereji et al. (2018), which was based on the H3Q85C cleavage method, we also performed the same analysis. The profiles were generally similar to those obtained based on the MNase-seq-based map. In this analysis, however, the asymmetry in the 3′-UTR profiles was more pronounced, confirming that the nucleosome occupancies change between upstream and downstream regions of the IRs in 3′-UTRs, from high to low. For the focused region in ORFs, the majority of the type VII IRs was also found within nucleosomes.

**Fig. 6:**
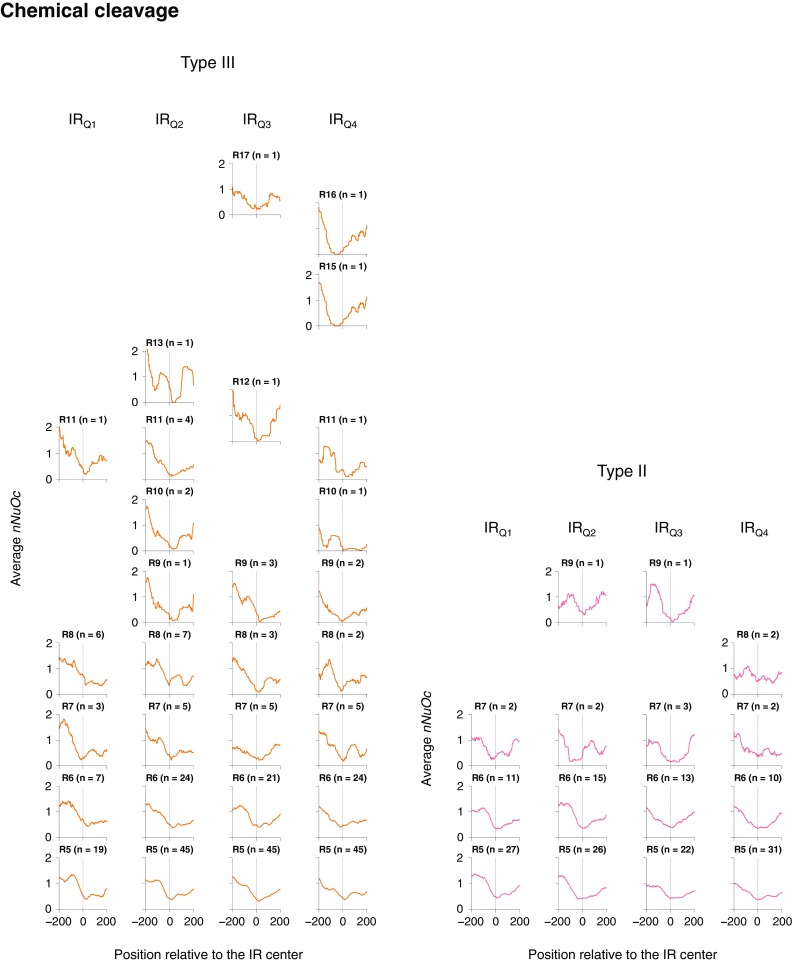
Relationship between the IR structure and the nucleosome occupancy. The IRs located in 3′-UTRs were sorted according to the repeat unit length, and the same analysis as in Fig. 5 was performed. For the data on nucleosome positions, only the chemical cleavage-based data (Chereji et al. 2018) were used in this analysis. ‘R5’–‘R17’, repeat unit lengths of 5–17 bp

Finally, we examined the relationship between the IR structure and the nucleosome occupancy for the IRs found in 3′-UTRs (Fig. 6). This analysis revealed several interesting points. Firstly, as the unit lengths of the type III IRs increased, the average values of the normalized nucleosome occupancies (*nNuOcs*; “Materials and methods”) decreased. Second, in most cases, when the length of the type III IRs becomes ≥ 9, the average *nNuOc* value becomes ~ 0 on the IRs or very close to them. In the latter cases, another IR or IRs or A/T-rich tracts were often found to be the sites of the ~ 0 value (Supplementary Fig. S1). Third, the type II IRs that showed values ~ 0 are rare (this may be caused by the lengths of their repeat units: those with R ≥ 10 were not found and 92% of them had a repeat unit length of 5–6 bp).

## Discussion

We performed genome-wide analyses for the distribution, occurrence frequency, sequence characteristics and relevance to chromatin structure of the IRs that reportedly have a cruciform-forming potential. The IRs are widely distributed in the yeast genome. The ApT- or TpA-rich type III IRs and A-tract- or T-tract-rich type II IRs are enriched in 3′-UTRs, especially in the close vicinity of poly(A) sites. The majority of these types is located in linker DNA regions. In the region ~ 30 to ~ 60 bp downstream of start codons, the type VII IRs, which are neither AT-rich, A- or T-tract-rich, nor (ApT)_*n*_ or (TpA)_*n*_-rich, are enriched and located within the + 1 nucleosome. In contrast, fewer IRs are present in the adjacent region downstream of start codons and around ~ 15 bp downstream of TSSs. Here, we discuss what these phenomena suggest with regard to the genetic events.

### What the positions and the types of IRs suggest

The types III and II IRs are enriched in 3′-UTRs. They seem to correspond to the important elements in RNA that are used as the poly(A) signal, PAS. Furthermore, the nucleosome occupancy changes within the 3′-UTR from high (upstream) to low (downstream), and these IRs are located at the border (Fig. 5). Although the PAS of *S. cerevisiae* is reportedly very degenerate and thus recognizing the PAS in a given gene is sometimes difficult, the current study provides a new perspective on this issue. Generally, from upstream to downstream, a PAS consists of an AU-rich efficiency element ‘EE’ (UAYRUA: Y = U or C, R = A or G), an A-rich positioning element ‘PE’ (AAWAAA: W = A or U) that is typically located ~ 10 to ~ 30 nucleotides upstream of the cleavage position, and a U-rich element spanning the cleavage position and the site of poly(A) addition (Guo and Sherman 1996; Zhao et al. 1999; Proudfoot 2011; Mischo and Proudfoot 2013). The EE and PE sequences seem to correspond to the types III and II IR sequences of the DNA, respectively. Furthermore, the mutual positional relationships among the EE, the PE and the site of poly(A) addition are very similar to those among the type III IR, the type II IR and the poly(A) site. The type II IRs occur slightly closer to the poly(A) sites than the type III IRs, in general. Thus, these analyses indicated that in a certain population of genes, the EE-coding DNA region and/or the PE-coding DNA region presumably constitute(s) the repeat units of the type III IRs and/or that of the type II IRs, respectively. Viewed in this light, the types III and II IRs seem to function at the RNA level, rather than the DNA level.

The type VII IRs are enriched in the regions ~ 30 to ~ 60 bp downstream of start codons. They are not AT-rich (the average AT content in the repeat units of the type VII IRs located in this region is ~ 40%) and lack the sequence advantage for cruciform formation, and are actually located within nucleosomes (Fig. 5). Thus, if they have some biological function, it would presumably be at the RNA level. The function may be some “riboregulator”-like one found in bacteria (Merino and Yanofsky 2005; Wachter 2014; Millman et al. 2017). The riboregulators can assume two mutually exclusive RNA structures in the primary transcripts: one forms a terminator and results in premature transcription termination, and the other forms an antiterminator that allows the production of a full-length mRNA by read-through into the coding sequence (Millman et al. 2017). Although riboregulator-related IRs usually occur in the 5′-UTR in *E. coli*, we suggested that such IRs may also occur in the region ~ 25 to ~ 60 bp downstream of the start codons in this organism (Miura et al. 2018). Furthermore, it must be noted that conditional transcriptional terminator-like structures, which have an IR followed by a U-rich tract, are sometimes found in the focused regions (data not shown). Thus, in *S. cerevisiae*, the IRs in the regions ~ 30 to ~ 60 bp downstream of start codons may play some riboregulator-like role.

We also found an IR-deficient region adjacent downstream of start codons (Fig. 2). Since a stem-loop RNA structure formed near a start codon would negatively influence translation initiation, this situation may be diminished in yeast. The region around ~ 15 bp downstream of TSSs was another site of low IR occurrence. For this phenomenon, we presently cannot give any plausible explanation.

### Possible causes of low nucleosome occupancy on the types III and II IRs

The region around ~ 100 bp downstream of a stop codon is known to have relatively low nucleosome occupancy in yeast (Kaplan et al. 2009; Pan et al. 2011). To explain this phenomenon, a hypothesis was raised that PASs disfavor nucleosome formation (Kaplan et al. 2009). This putative propensity of PASs may be caused by the types III and II IRs in a certain population of genes. For the type III IRs, the cruciform formation is the first issue to discuss as a possible cause. Dayn et al. (1991, 1992) reported that all detected in vivo cruciforms are formed by AT-rich inverted repeats, particularly (ApT)_*n*_ sequences. Other groups also arrived at similar conclusions (McClellan et al. 1986, 1990; Panayotatos and Fontaine 1987; Wells and Harvey 1987; Horwitz and Loeb 1988; Calladine et al. 2004). Mechanistically, the very small contribution of the stacking forces of the (ApT)_*n*_ sequences to stabilize the B-form is likely to be the cause of the transition into cruciforms (Panayotatos and Fontaine 1987). However, the hypothesis of “B to cruciform transition” for the type III IRs has a “size-problem”.

The size of a cruciform is a debated issue. Vologodskii et al. suggested that cruciform extrusion in short palindromes with low supercoiling is highly improbable (Vologodskaia and Vologodskii 1999; Vologodskii 2015), and a theoretical study by Zhabinskaya and Benham (2013) was in accordance with this suggestion. In the latter study, the cruciforms with stem lengths of < ~ 15 bp seemed improbable (however, DNA melting seemed possible even for the IRs with ~ 3 bp repeats). In the current study, short IRs with repeat units of < ~ 15 bp were found to be the majority, including the type III IRs, in the yeast genome (Figs. 1, 6, http://www.waseda.jp/sem-ohyama/CFIRs-Sc). Thus, based on the studies by Vologodskii et al. and Zhabinskaya and Benham, the in vivo transition of the type III IRs into cruciforms may be “highly improbable” (but melting or deformation seems possible). However, we must also note that numerous reports have shown or proposed the presence of cruciforms with short stems of 5–7 bp (Sheflin and Kowalski 1985; Iacono-Connors and Kowalski 1986; Müller and Wilson 1987; McMurray et al. 1991; Dai et al. 1997; Dai and Rothman-Denes 1998; Jagelská et al. 2010; Nuñez et al. 2015). Based mainly on the latter reports, the current study regarded the R ≥ 5S ≤ 8 (2R + S ≥ 13) IRs as those that have a “potential” for transition into cruciforms. Importantly, this does not mean that they are actually forming cruciforms in vivo or have “high” potential for cruciform formation. The level of the potential was not the point in the current study.

The focus here is what causes the low nucleosome occupancy on the type III IRs. We found that the average *nNuOc* values decrease, even to 0, according to the increase of the repeat unit length of the type III IRs (Fig. 6). This phenomenon seems to be explained in terms of the increase of deformed B-form structures or cruciform occurrence. Although the occurrence of these non-B structures may be transient, even for the larger type III IRs, it may be sufficient to exclude nucleosomes. The presence of multiple IRs or A/T-rich tracts in a small region may increase these probabilities overall (Supplementary Fig. S1). However, at present, we cannot still deny the formation of “stable” cruciforms in some cases. Some unknown effect only seen in vivo, including dynamic genetic processes that can locally generate a high density of negative supercoiling temporarily or even a simple loss of nucleosomes may be able to generate the cruciforms with short stems. Finally, we must also discuss the possibility for the formation of alternative structures. The (ApT)_*n*_ tracts can also form Z-DNA structures. However, this seems to be less probable. The propensity for forming Z-DNA is in the following order: (GpC)_*n*_ > (CpA)_*n*_ > (CGGG)_*n*_ > (ApT)_*n*_ (Wang et al. 1984; Shin et al. 2016). Furthermore, it is known that the (ApT)_*n*_ tracts more prefer cruciform formation than Z-DNA formation (Wang et al. 1984, 2013; Sinden 1994). In summary, (transient) deformation or cruciform formation is raised as a possible mechanism underlying the low nucleosome occupancy on or around the type III IRs.

For the A-tract- or T-tract-rich type II IRs, the low nucleosome occupancy may be caused by different mechanisms. The A/T-tracts and oligo(A/T) sequences are reportedly rigid (Nelson et al. 1987; Packer et al. 2000; Suter et al. 2000), and seem to resist bending around the histone core (Iyer and Struhl 1995; Segal and Widom 2009; Struhl and Segal 2013). Indeed, a genome-scale analysis for nucleosome positions showed that these sequences are usually not incorporated into nucleosomes (Yuan et al. 2005). Furthermore, intrinsically bent DNA structures, which can either inhibit or facilitate nucleosome formation due to the 3D structure (Ohyama 2001), may also be partly relevant. These structures are formed under the following conditions: an A- or T-tract is present within the spacer region in a given IR in phase with the tracts within the two repeat regions, or A- or T-tracts accidentally occur in the flanking regions of a given IR in phase with the tracts inside the IR. In the case where the periodicity of the tract is ≥ 11 bp, an unfavorable 3D structure for nucleosome formation is generated. Indeed, such cases are sometimes found in the type II IRs focused upon here (data not shown). However, we should also note the report by Kornberg’s group. They found that the nucleosome-free regions are formed and maintained by an active mechanism involving chromatin remodeling, with RSC (the most abundant member of the SWI/SNF family) recognition of T-tract-rich sequences, rather than the DNA rigidity- or conformation-based mechanism described above (Lorch et al. 2014). Considering these possibilities, several A-tract- or T-tract-originated mechanisms other than cruciform formation are likely to cause the low nucleosome occupancy on the type II IRs. Thus, the mechanistic cause for the low nucleosome occupancy seems to be essentially different between the types III and II IR sequences.

In addition to the types III and II sequences and the putative action of RSC, the dynamic migration of RNA polymerase II (pol II) may also contribute to the low nucleosome occupancy. The rapid removal of pol II reportedly causes increased nucleosome occupancy around poly(A) sites (Fan et al. 2010). Thus, the dynamic changes in the superhelical state caused by transcription, pol II migration itself, some action by the RSC, and the intrinsic properties and/or conformations of the type III and II IRs may collaborate with one another and induce the nucleosome depletion.

### Similarity in the IR occurrence between *E. coli* and *S. cerevisiae*

**Fig. 7:**
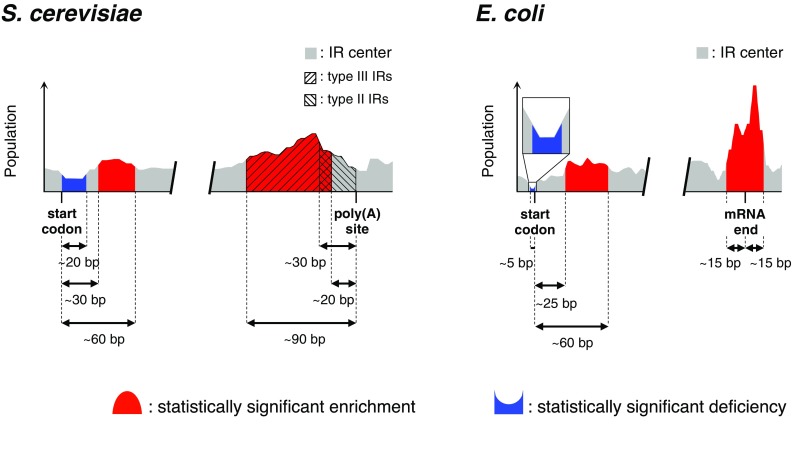
Similarity in the IR occurrence between *E. coli* and *S. cerevisiae*. The illustration for *E. coli* is based on Miura et al. (2018) and that for yeast is based on the data shown in Fig. 2 (‘3′-UTR’ and ‘ORF’ panels). The ‘mRNA end’ in the *E. coli* illustration indicates the experimentally determined position, and the actual end position seems to be located farther downstream relative to the shown IR peak (Miura et al. 2018)

The genomes of *E. coli* and *S. cerevisiae* have two common regions with statistically significant enrichment of IRs: one is in the close vicinity of the positions corresponding to mRNA ends (*E. coli*; Miura et al. 2018) or poly(A) sites (*S. cerevisiae*) and the other is ~ 25 to ~ 60 bp (*E. coli*; Miura et al. 2018) or ~ 30 to ~ 60 bp (*S. cerevisiae*) downstream of the start codons (Fig. 7). For the former, most of the IRs in *E. coli* seem to be used as parts of intrinsic terminators and they are GC-rich (Miura et al. 2018). In contrast, the IRs in *S. cerevisiae* seem to function as parts of the PAS signal and they are AT-rich, as described above. Thus, the *E. coli* and *S. cerevisiae* IRs both seem to function at the RNA level in each transcription termination system, although their nucleotide compositions are quite different. The differences in the DNA sequences may originate from the absence or presence of chromatin structure. In the case of *S. cerevisiae*, the IRs are also used to decrease nucleosome occupancy at the DNA level and for this purpose, A- or T-tract-rich, or (ApT)_*n*_ or (TpA)_*n*_-rich IRs are favorable, as described above.

For the regions in ORFs, the similarity between the two organisms also alludes to the presence of some common role of the IRs, which is presumably played at the RNA level. Furthermore, it is notable that the IRs with cruciform-forming potential are actively excluded in the translation initiation regions, not only in *S. cerevisiae* but also in *E. coli* (Miura et al. 2018). From this viewpoint, we can safely conclude that the IRs presumably play similar roles in the prokaryote *E. coli* and the lower eukaryote *S. cerevisiae* to regulate or complete transcription at the RNA level.

## Electronic supplementary material

Below is the link to the electronic supplementary material.

Supplementary material 1 (PDF 392 KB)
