# Supplementary material for: A strong structural correlation between short inverted repeat sequences and the polyadenylation signal in yeast and nucleosome exclusion by these inverted repeats
Source: Curr Genet. 2018 Nov 29;65(2):575–90. doi: 10.1007/s00294-018-0907-8 (PMC6420913; doi:10.1007/s00294-018-0907-8)
Supplement: Supplementary file 1 — Supplementary material 1 (PDF 392 KB) [file 294_2018_907_MOESM1_ESM.pdf]

A strong structural correlation between short inverted repeat sequences and the polyadenylation signal in yeast and nucleosome exclusion by these inverted repeats

Current Genetics

Osamu Miura[1], Toshihiro Ogake[2], Hiroki Yoneyama[2], Yo Kikuchi[2] and Takashi Ohyama[1,2,*]

[1] Department of Biology, Faculty of Education and Integrated Arts and Sciences, Waseda University, 2-2 Wakamatsu-cho, Shinjuku-ku, Tokyo 162-8480, Japan

[2] Major in Integrative Bioscience and Biomedical Engineering, Graduate School of Science and Engineering, Waseda University, 2-2 Wakamatsu-cho, Shinjuku-ku, Tokyo 162-8480, Japan

* To whom correspondence should be addressed. Tel: +81 3 5369 7310; Fax: +81 3 3355 0316; Email: ohyama@waseda.jp

Supplementary Table S1

Supplementary Table S1 The average size of each region.

Region	Count	Average size (bp)
5'-UTR	4,501	86
ORF	4,370	1,536
3'-UTR	3,172	130
OUR-1 (5’-UTR & 5’-UTR)	37	143
OUR-2 (3’-UTR & 5’-UTR)	174	199
OUR-3 (3’-UTR & 3’-UTR)	882	177
TAN	1,829	305
DIV	990	420
CON	379	209

Supplementary Fig. S1

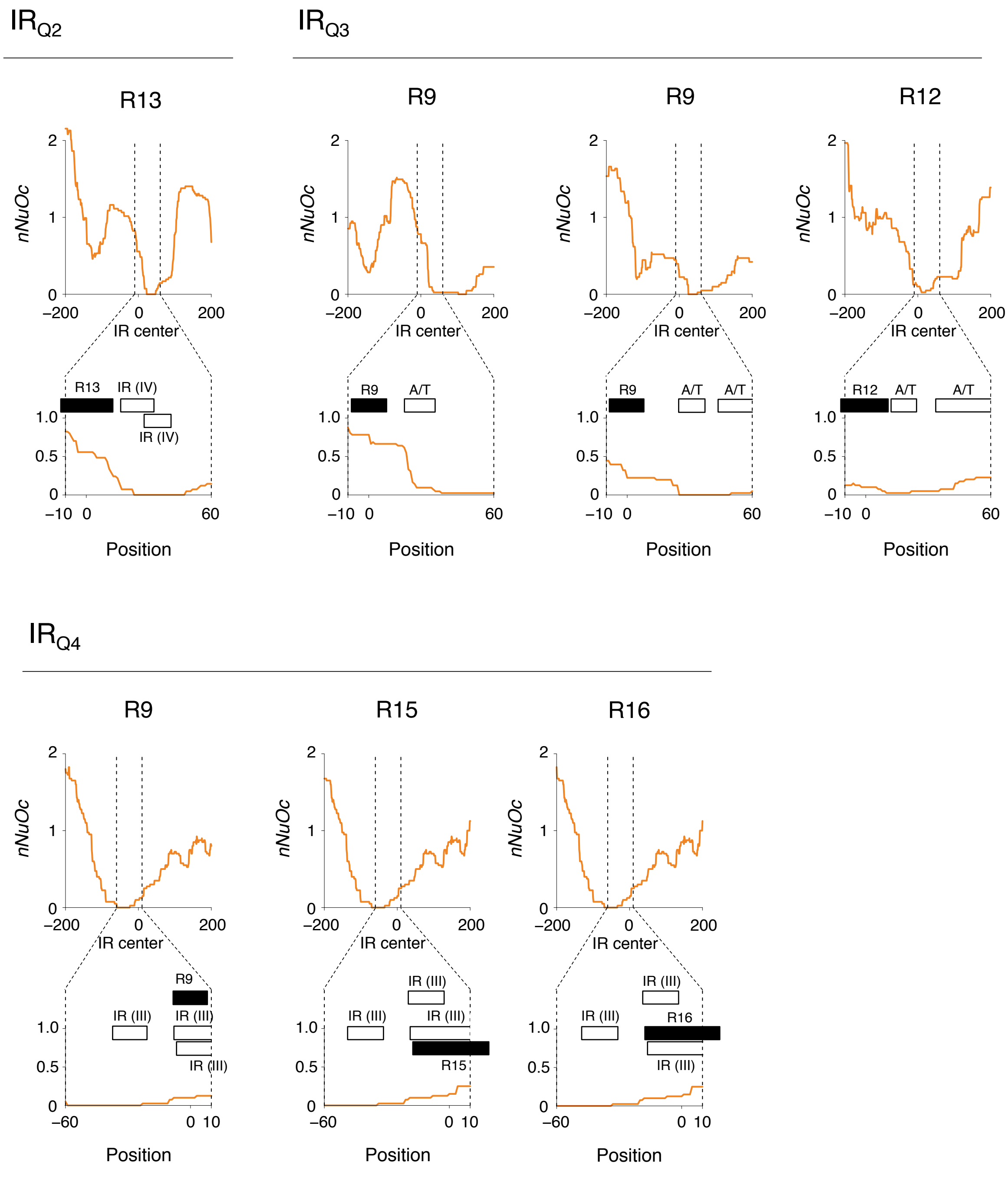

Supplementary Fig. S1 The *nNuOc* value of zero is often caused by the collaboration with neighboring IRs or A/T-rich tracts. The IRs with the structure R≥9S≤8 account for 20. Among them, 16 showed the *nNuOc* value of ~0 on or around the IRs. Furthermore, among the 16 IRs, five (5/16 = 31%) correspond to the bottom of the *nNuOc* profile (position of the value ~0) and 11 have the bottom adjacent to them. Among these 11 IRs, four (4/11 = 36%) flank the other IR(s), three (3/11 = 27%) flank A/T-rich tracts and four flank sequences without any specific motifs, and these correspond to the sites with values ~0. All of the *nNuOc* profiles of the 7 IRs (four that flank the other IR(s) and three that flank A/T-rich tracts) are shown. Filled bar, focused IR in Fig. 6. Open bar, neighboring IR (type is shown in parentheses) or A/T-rich tracts.
